# Therapeutic Effect of Exendin-4, a Long-Acting Analogue of Glucagon-Like Peptide-1 Receptor Agonist, on Nerve Regeneration after the Crush Nerve Injury

**DOI:** 10.1155/2013/315848

**Published:** 2013-08-04

**Authors:** Koji Yamamoto, Masatoshi Amako, Yoritsuna Yamamoto, Toyokazu Tsuchihara, Hitoshi Nukada, Yasuo Yoshihara, Hiroshi Arino, Masanori Fujita, Maki Uenoyama, Shoichi Tachibana, Koichi Nemoto

**Affiliations:** ^1^Department of Orthopedic Surgery, National Defense Medical College, 3-2 Namiki, Tokorozawa, Saitama 359-8513, Japan; ^2^Division of Environmental Medicine, National Defense Medical College Research Institute, 3-2 Namiki, Tokorozawa, Saitama 359-8513, Japan; ^3^Orthopedic Surgery, Japan Self Defense Forces Central Hospital, 1-2-24 Ikejiri, Setagaya-ku, Tokyo 154-0001, Japan; ^4^Department of Medicine, University of Otago Medical School, P.O. Box 913, Dunedin 9054, New Zealand; ^5^The Nukada Institute for Medical and Biological Research, 5-18 Inage-cho, Inage-ku, Chiba 263-0035, Japan

## Abstract

Glucagon-like peptide-1 (GLP-1) is glucose-dependent insulinotropic hormone secreted from enteroendocrine L cells. Its long-acting analogue, exendin-4, is equipotent to GLP-1 and is used to treat type 2 diabetes mellitus. In addition, exendin-4 has effects on the central and peripheral nervous system. In this study, we administered repeated intraperitoneal (i.p.) injections of exendin-4 to examine whether exendin-4 is able to facilitate the recovery after the crush nerve injury. Exendin-4 injection was started immediately after crush injury and was repeated every day for subsequent 14 days. Rats subjected to sciatic nerve crush exhibited marked functional loss, electrophysiological dysfunction, and atrophy of the tibialis anterior muscle (TA). All these changes, except for the atrophy of TA, were improved significantly by the administration of exendin-4. Functional, electrophysiological, and morphological parameters indicated significant enhancement of nerve regeneration 4 weeks after nerve crush. These results suggest that exendin-4 is feasible for clinical application to treat peripheral nerve injury.

## 1. Introduction

Peripheral nerve injury is common in the clinical field and the prevalence is relatively high (2.8% of trauma patients) [1]. However, its prognosis is often unsatisfactory because nerve regeneration is slow and it takes long time to reinnervate the target organs. As a result, some functional and histological changes become irreversible. Therefore, it is important to accelerate nerve regeneration to obtain excellent recovery.

GLP-1 is glucose-dependent insulinotropic hormone secreted from enteroendocrine L cells [2]. However, its therapeutic potential in diabetes is limited by its short biological half-life through degradation by dipeptidyl peptidase IV. Exendin-4 is a GLP-1 analogue derived from the saliva of the glia monster that shares 53% sequence homology with GLP-1 [3, 4]. It is equipotent to GLP-1 and has longer half-life than GLP-1 due to resistance to degradation by dipeptidyl peptidase IV [5]. Therefore, exendin-4 is approved to improve glycemic control in patients with type 2 diabetes mellitus.

GLP-1 and exendin-4 also have effects on the central and peripheral nervous system. GLP-1 and exendin-4 have been shown to have protective effect to the central nervous system from neurodegenerative disease [6–8]. GLP-1 and exendin-4 attenuate the peripheral sensory neuropathy induced by pyridoxine [9]. GLP-1 also promotes neurite outgrowth in PC12 cells *in vitro* [10]. However, to our knowledge, there is no demonstrated data of exendin-4 in the peripheral nerve injury. In this study, we examined whether exendin-4 can enhance functional and histological recovery after crush injury of the rat sciatic nerve.

## 2. Materials and Methods

### 2.1. Animals

Forty-eight Wistar rats, 8 weeks old and weighing 175–210 g, were used in all experiments. Animals were obtained from Japan SLC, Inc. (Hamamatsu, Japan). They were housed in a temperature-controlled room with a 12-hour light-dark cycle. 

### 2.2. Design of Animal Experiments

All surgical procedures were carried out as described previously [11]. Rats were divided randomly into 4 equal groups as follows: (1) crush group (*n* = 12), administered 0.5 mL saline/rat/day after a 5 min. nerve crush injury, (2) crush + exendin-4 group (*n* = 12), administered 2.5 *μ*g/rat/day exendin-4 (Sigma-Aldrich, St Louis, MO, USA) in 0.5 mL saline starting after 5 min. of nerve crush injury, (3) exendin-4 group (*n* = 12), administered 2.5 *μ*g/rat/day exendin-4 in 0.5 mL saline only without operation, and (4) sham operation group (*n* = 12), administered 0.5 mL saline/rat/day after sham operation with no crush injury. Injections of exendin-4 or saline were done from 1 to 14 days after the crush. 

### 2.3. Evaluation

#### 2.3.1. Sciatic Functional Index (SFI)

For the evaluation of motor function, walking tracks were recorded as previously mentioned [12]. The following measurements were taken: the distance from heel to toe (the print length, PL), the distance between the first and fifth toes (the toe spread, TS), and the distance from the second to the fourth toes (the intermediary toe spread, IT). SFIs were calculated as previously described [13]. Assessments were performed at 11 defined time points and up to 28 days after surgery (*n* = 12 in each group).

#### 2.3.2. Glucose Measurement

Blood samples were obtained from the tail vein, and glucose levels were measured using a blood glucose meter (FreeStyle; Nipro Inc, Osaka, Japan) at 0 (before crush), 1, and 3 days after crush (*n* = 6 in each group). 

#### 2.3.3. Electrophysiological Study

At 4 weeks after the crush, the right sciatic nerve was exposed under sodium pentobarbital anesthesia (40–50 mg/kg, i.p.), and the nerve was stimulated at 2 mm distal to the sciatic notch, using a hook-up electrode. Evoked muscle response was recorded from TA using needle electrodes. We kept the distance between the stimulating and recording sites as 42 mm, which was measured on the surface of the fully extended hind limbs. Both compound muscle action potential (CMAP) and distal latency were measured using Neuropack instrumentation (Nihon Kohden inc, Tokyo, Japan) (*n* = 6 in each group).

#### 2.3.4. Assessment of the Wet Weight of TA

At 4 weeks after the crush, both TA were harvested and weighed as previously described [14] (*n* = 12 in each group).

#### 2.3.5. Histological Studies of the Sciatic Nerve

Specimens of the crushed sciatic nerve were taken at 5 mm distal to the crush site after 4 weeks (*n* = 6 in each group). All specimens were fixed in 2.5% glutaraldehyde in 0.1 M phosphate buffer solution (pH 7.4) overnight. Subsequently, they were postfixed with 1% osmium tetroxide, dehydrated, and cut into consecutive 2-3 mm blocks before embedding in Epon resin (EPON 812, TAAB Ltd, Berkshire, England).

*Light Microscopic Study*. Semi-thin sections (1 *μ*m in thickness) of sciatic nerves were stained with toluidine blue and were examined by light microscope. The area and the number of the myelinated axons were measured as described previously [15–17]. At least, 1400 myelinated fibers were counted in each nerve. 

*Electron Microscope*. Thin sections (60 nm in thickness) of sciatic nerves were stained with uranyl acetate and citrate and were examined by electron microscope. The *g*-ratios of myelinated axons were calculated in 12 different fields of each section and analyzed [18]. In average, we counted 140 myelinated fibers in each nerve.

#### 2.3.6. Statistical Analysis

Statistical analyses were performed using JMP 10 (SAS Institute Inc., Cary, NC, USA). We expressed values for each parameter as means ± SD. *P* values of <0.05 were considered significant. Two-way analysis of variance (ANOVA) was utilized to examine the main effects of the nerve crush (with crush versus without crush) and exendin-4 administration (exendin-4 injection versus saline injection). Tukey test was run for post hoc analysis.

### 2.4. Ethics

This experimental study was carried out in accordance with the recommendations in the Guide for the Care and Use of Laboratory Animals published by the National Institutes of Health, and the protocol was approved by the Committee on the Ethics of Animal Experiments of the National Defense Medical College (no. 11102).

## 3. Results

### 3.1. Sciatic Functional Index (SFI)

**Figure 1:**
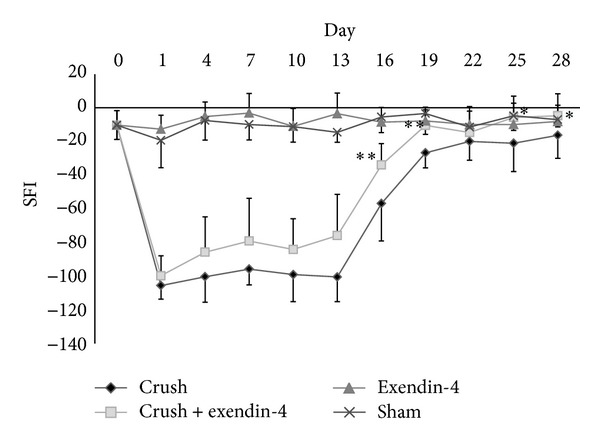
Sciatic functional index. SFI was calculated by the following formula [13]: SFI = −38.3(EPL − NPL)/NPL + 109.5(ETS − NTS)/NTS + 13.3(EIT − NIT)/NIT−8.8. E, and N represent experimental and normal sides, respectively. In the crush + exendin-4 group, the SFI recovered rapidly and returned to the precrush levels by 19 days although in the crush group it did not return to the presurgical level at the end of the study. At 16, 19, 25, and 28 days after the crush injury, the SFIs in the crush + exendin-4 group were significantly higher than those in the crush group (*P < 0.05, **P < 0.01 compared with crush group).

In the crush and crush + exendin-4 groups, the SFIs decreased from levels near −10 (representing normal function) to levels near −100 (representing complete loss of function) after surgery. In the crush + exendin-4 group, the SFI recovered rapidly and returned to the precrush levels by 19 days although in the crush group it did not return to the presurgical level at the end of the study. In the sham operation and exendin-4 groups, the SFIs did not alter significantly through this study. At 16, 19, 25, and 28 days after the procedure, two-way ANOVA revealed significant interaction between the main effects of the nerve crush and exendin-4 administration. The post hoc data indicated that the SFIs in the crush + exendin-4 group were significantly higher than those in the crush group (Figure 1).

### 3.2. Glucose Measurement

In all groups, the blood glucose levels were normal at 0 (before crush), 1, and 3 days after the nerve crush.

### 3.3. Electrophysiological Study

The CMAP was increased in the crush + exendin-4 group, compared with that in the crush group at 4 weeks after the crush. However, no interaction (*P* = 0.148) between the main effects of the nerve crush and exendin-4 administration was found. With regard to the distal latency, two-way ANOVA showed that the effects of nerve crush and exendin-4 administration were affected significantly (*P* < 0.01). Interaction between these two factors was significant (*P* < 0.01). The post hoc data showed that the distal latency in the crush group was significantly shorter than that in the crush + exendin-4 group (*P* < 0.01) (Table 1).

### 3.4. Wet Weight of TA

In the crush and crush + exendin-4 groups, the TAs on the injured side were atrophic, while the TAs on the uninjured side were normal. Two-way ANOVA indicated that the TA ratio (injured side/uninjured side) was affected by the effects of nerve crush (*P* < 0.01) but not affected by exendin-4 administration (*P* = 0.23). There was no interaction (*P* = 0.06) between these two effects (Table 2). 

### 3.5. Histological Studies of the Sciatic Nerve

#### 3.5.1. Light Microscopic Studies

**Figure 2:**
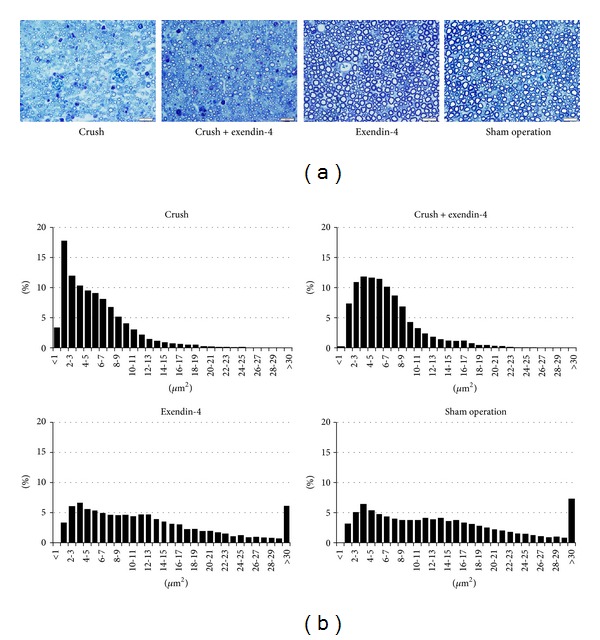
Light microscopic findings of the sciatic nerve. (a) Semi-thin transverse sections of myelinated axons of the sciatic nerve at 4 weeks postoperatively. In the crush + exendin-4 group, regenerating axons were more mature than those in the crush group. Scale bar: 20 *μ*m. (b) Histograms of areas of myelinated axons (*n* = 6 in each group). Compared with the crush group, the peak of histogram in crush + exendin-4 group shifted to the right which indicates the maturation of regenerating fibers (Figure 2(b)).

In the crush and crush + exendin-4 groups, degenerating debris and small axons with a thin myelin sheath were prominent at 4 weeks after surgery. In the crush + exendin-4 group, regenerating axons were more mature than those in the crush group. Specimens taken from the sham operation and exendin-4 groups showed dense axon populations with large axons and thick myelin sheaths. In the sham operation and exendin-4 groups, nerve pathology was normal (Figure 2(a)). A histogram of myelinated axon areas in the sham operation group showed a bimodal distribution: small fibers peak at 3.5 *μ*m^2^ and large fibers at 12.5 *μ*m^2^. In crush and crush + exendin-4 groups, the number of small fibers increased dramatically, resulting from regenerating fibers. Compared with the crush group, the peak of histogram in crush + exendin-4 group shifted to the right which indicates the maturation of regenerating fibers (Figure 2(b)). Analyses to examine the effects of nerve crush and exendin-4 administration on mean axon areas and mean axon numbers were conducted using two-way ANOVA. These results showed that nerve crush has significant effect on both mean axon areas and mean axon numbers (*P* < 0.01) but exendin-4 administration neither has effect on axon areas (*P* = 0.849) nor axon numbers (*P* = 0.589). The interaction between these two effects was significant on both axon areas (*P* < 0.05) and axon numbers (*P* < 0.01) (Table 3).

#### 3.5.2. Electron Microscopic Studies

**Figure 3:**
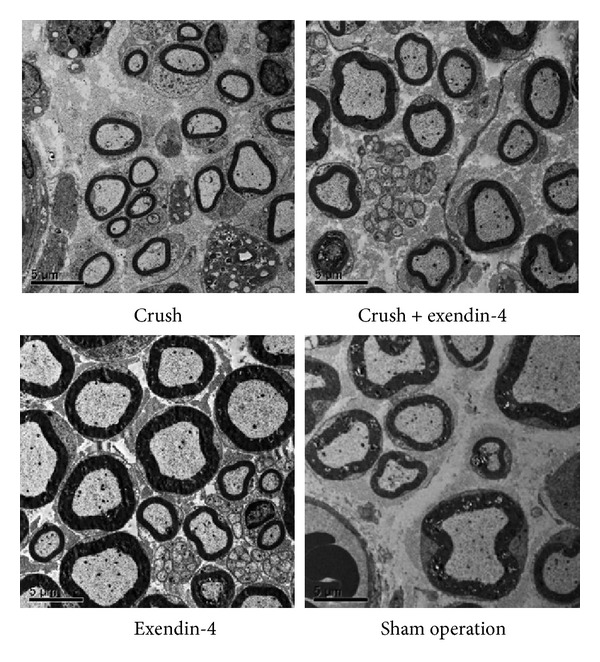
Electron microscopic findings of the sciatic nerve. Thin sections (60 nm in thickness) of sciatic nerve at 4 weeks postoperatively, stained with uranyl acetate and citrate. In the crush + exendin-4 group, the thickness of the myelin sheath was slightly greater than that in the crush group. Scale bar: 5 *μ*m.

 In both the crush and crush + exendin-4 groups, the thickness of the myelin sheath was thinner than that in the sham operation group. In the crush + exendin-4 group, the thickness of the myelin sheath was slightly greater than that in the crush group (Figure 3). Two-way ANOVA indicated that the *g*-ratio was affected by the effects of nerve crush (*P* < 0.01) but not affected by exendin-4 administration (*P* = 0.71). Interaction between these two factors was significant (*P* < 0.05) (Table 4).

## 4. Discussion

Exendin-4 is a good candidate as therapeutic agent that facilitates nerve regeneration because of the potency for both the central and peripheral nervous system [6, 8–10, 19] and has been used for patients with type 2 diabetes mellitus in clinical field. In this study, we examined whether exendin-4 can enhance functional and histological recovery after crush injury of the rat sciatic nerve.

 We demonstrated that administration of exendin-4 was effective in the crush + exendin-4 group compared with the crush group. (1) The entire SFI curve was shifted to the left, which suggests facilitation of nerve regeneration. (2) In the electrophysiological study, shortening of the mean latency was demonstrated. (3) In light microscopic findings, nerve regeneration was enhanced by the administration of exendin-4. (4) The morphometrical findings of light microscopic study showed enhancement of nerve regeneration. (5) The *g*-ratio was significantly lower. Together, these results suggest that the administration of exendin-4 accelerated regeneration of the injured nerve and final functional recovery.

In the current study, we adopted the nerve crush model to evaluate the effectiveness of exendin-4. Since it represents an axonotmesis-type injury [20–22] in which the basal membrane of Schwann cells is maintained, it prevents misdirection, and functional recovery occurs relatively quickly. In nerve transection models, it is difficult to obtain sufficient functional recovery within a year because of misdirection and chronic joint contracture [23]. 

The doses of exendin-4 vary among animal studies: 10 *μ*g/mouse to reduce the size of cerebral infarction [24] and, 3.5 pM/kg/min for neuroprotection of peripheral nerves [5, 9, 10]. In this study, we administered 2.5 *μ*g/rat/day exendin-4 for 14 days, which is the highest total dose compared with previous studies. Low-dose administrations of exendin-4 are effective mainly for chronic conditions such as Alzheimer's disease or DM neuropathy, while high-dose administration of exendin-4 is required in acute injury.

Exendin-4, a long-acting analogue of GLP-1, can improve hyperglycemic states [7] and can inhibit major complications of diabetes mellitus, such as neuropathy, nephropathy, or retinopathy [25–27]. In the present study, the levels of blood glucose were normal in all groups. Studies showed that the administration of exendin-4 exerted neuroprotection for peripheral sensory neuropathy induced by pyridoxine [9] and transient cerebral focal cerebral ischemia [24]. Jolivalt et al. [28] mentioned that exendin-4 could protect peripheral nerves by the GLP-1 receptor (GLP-1R) activation mediated by ERK signaling independent of glycemic control. Liu et al. [25] also noted that exendin-4 may prevent peripheral nerve degeneration by the activation of GLP-1R, antiapoptotic effects, and restoration of cyclic adenosine monophosphate content rather than an improvement of blood glucose. 

In peripheral nerve regeneration, remyelination of the regenerated axons is required [29], and Schwann cells are responsible for the remyelination [30]. Previous studies demonstrated that GLP-1Rs were expressed in the Schwann cells of the sciatic nerve and activated by exendin-4 [25, 28]. These results suggest that, in the crushed nerve, exendin-4 encourages peripheral nerve regeneration through activation of Schwann cells mediated by GLP-1Rs.

In conclusion, repeated i.p. injections of exendin-4 can promote nerve regeneration and functional recovery after nerve crush injury. Therefore, exendin-4 is feasible for clinical application to treat peripheral nerve injury.

## Figures and Tables

**Table 1:** Electrophysiological study at 4 weeks after crush (*n* = 6 in each group).

Group	CMAP	Latency
	Mean [SD] (mV)	Mean [SD] (ms)
Crush	8.38 [2.43]	3.53 [0.31]
Crush + exendin-4	11.87 [1.56]	2.87 [0.46]**
Exendin-4	15.30 [4.69]	1.46 [0.10]**
Sham operation	16.22 [4.59]	1.45 [0.09]**

**P < 0.01 compared with crush group.

**Table 2:** Weight ratio of the TA (*n* = 12 in each group).

Group	Ratio
	Mean [SD]
Crush	0.79 [0.05]
Crush + exendin-4	0.73 [0.09]
Exendin-4	1.01 [0.05]
Sham operation	1.02 [0.06]

**Table 3:** Mean axon areas and numbers of myelinated axon at 4 weeks after nerve crush (*n* = 6 in each group).

Group	Axon area	Axon number
	Mean [SD] (*μ*m^2^)	Mean [SD]
Crush	5.67 [0.72]	1950.6 [162.6]
Crush + exendin-4	6.57 [0.86]	1736.1 [203.1]
Exendin-4	12.77 [1.09]	1774.6 [114.2]
Sham operation	13.84 [1.22]	1611.5 [125.9]

**Table 4:** *g*-ratio of the right sciatic nerves (*n* = 6 in each group).

Group	*g*-ratio
	Mean [SD]
Crush	0.71 [0.01]
Crush + exendin-4	0.69 [0.02]
Exendin-4	0.64 [0.01]
Sham operation	0.63 [0.02]

## Abbreviations

GLP-1: Glucagon-like peptide-1
GLP-1R: Glucagon-like peptide-1 receptor
i.p.: Intraperitoneal
TA: Tibialis anterior muscle
SFI: Sciatic functional index
CMAP: Compound muscle action potential
ANOVA: Analysis of variance.
